# Electroencephalographic β‐band oscillations in the sensorimotor network reflect motor symptom severity in amyotrophic lateral sclerosis

**DOI:** 10.1111/ene.16201

**Published:** 2024-01-18

**Authors:** Stefan Dukic, Antonio Fasano, Amina Coffey, Teresa Buxó, Roisin McMackin, Rangariroyashe Chipika, Mark Heverin, Peter Bede, Muthuraman Muthuraman, Madeleine Lowery, Richard G. Carson, Orla Hardiman, Bahman Nasseroleslami

**Affiliations:** ^1^ Academic Unit of Neurology, School of Medicine, Trinity College Dublin University of Dublin Dublin Ireland; ^2^ Department of Neurology, University Medical Centre Utrecht Brain Centre Utrecht University Utrecht the Netherlands; ^3^ Discipline of Physiology, School of Medicine, Trinity College Dublin University of Dublin Dublin Ireland; ^4^ Computational Neuroimaging Group, Trinity Biomedical Sciences Institute, Trinity College Dublin University of Dublin Dublin Ireland; ^5^ Neural Engineering With Signal Analytics and Artificial Intelligence, Department of Neurology University of Würzburg Würzburg Germany; ^6^ School of Electrical and Electronic Engineering University College Dublin Dublin Ireland; ^7^ Trinity College Institute of Neuroscience and School of Psychology, Trinity College Dublin University of Dublin Dublin Ireland; ^8^ School of Psychology Queen's University Belfast Belfast Ireland; ^9^ Department of Neurology Beaumont Hospital Dublin Ireland

**Keywords:** amyotrophic lateral sclerosis, biomarker, electroencephalography

## Abstract

**Background and purpose:**

Resting‐state electroencephalography (EEG) holds promise for assessing brain networks in amyotrophic lateral sclerosis (ALS). We investigated whether neural β‐band oscillations in the sensorimotor network could serve as an objective quantitative measure of progressive motor impairment and functional disability in ALS patients.

**Methods:**

Resting‐state EEG was recorded in 18 people with ALS and 38 age‐ and gender‐matched healthy controls. We estimated source‐localized β‐band spectral power in the sensorimotor cortex. Clinical evaluation included lower (LMN) and upper motor neuron scores, Amyotrophic Lateral Sclerosis Functional Rating Scale–Revised score, fine motor function (FMF) subscore, and progression rate. Correlations between clinical scores and β‐band power were analysed and corrected using a false discovery rate of *q* = 0.05.

**Results:**

β‐Band power was significantly lower in people with ALS than controls (*p* = 0.004), and correlated with LMN score (*R* = −0.65, *p* = 0.013), FMF subscore (*R* = −0.53, *p* = 0.036), and FMF progression rate (*R* = 0.52, *p* = 0.036).

**Conclusions:**

β‐Band spectral power in the sensorimotor cortex reflects clinically evaluated motor impairment in ALS. This technology merits further investigation as a biomarker of progressive functional disability.

## INTRODUCTION

Amyotrophic lateral sclerosis (ALS) is a multinetwork neurological disorder with impairment in the upper and lower motor neurons, as well as in the cognitive and behavioural systems [1]. Whereas the diagnosis is primarily based on clinical examination as well as on electromyographic findings, clinical trial outcome measures rely on semiquantitative tools, such as the Amyotrophic Lateral Sclerosis Functional Rating Scale–Revised (ALSFRS‐R). A consensus on ALS clinical trials has emphasized the necessity for easily measured, objective biomarkers [2] that quantify the progression of specific aspects of motor or cognitive decline.

A network‐based approach using neurophysiological techniques has promise in the development of biomarker candidates in neurodegenerative diseases [3] including ALS [4, 5]. Spectral electroencephalographic (EEG) measures have an excellent temporal resolution, as they capture electrical activity generated in the brain, whereas the limited spatial resolution can now be ameliorated using high‐density EEG and source‐localization techniques [6]. Consequently, more spatially precise and sensitive network interrogation methods can now be used in clinical settings [7]. Biomarkers based on resting‐state EEG have recently shown promise for interrogating multiple networks and for discovering complex ALS phenotypes [4, 8]. The β‐band spectral power in the sensorimotor network is shown to play an important role in ALS [8, 9, 10]. These oscillations are predominantly observed in the sensorimotor cortices and are underpinned by the corticospinal projections from the motor system to the muscles, representing a measure of the overall motor activity [11].

The utility of EEG‐based measures for specific assessment of progressive motor impairment and functional disability in people with ALS has not yet been adequately established. We hypothesized that β‐band oscillations in the cortical sensorimotor network can quantify the severity and progression of motor symptoms in ALS. We correlated these EEG‐based changes with clinical scores. Here, we provide evidence that the β‐band spectral power differs between people with ALS and healthy controls and correlates with progressive motor impairment and functional disability in ALS.

## METHODS

### Participants

The study was approved by the Tallaght University Hospital/St. James's Hospital Joint Research Ethics Committee Dublin (reference: 2019–05 List 17 [01]) and performed in accordance with the World Medical Association Declaration of Helsinki. All participants provided informed written consent and were prospectively recruited from the multidisciplinary ALS clinic based in Beaumont Hospital (Dublin, Ireland), and those in the ALS cohort were all diagnosed with definite/probable/possible ALS in accordance with the El Escorial Revised Criteria. Participants diagnosed with ALS restricted phenotypes or suffering from any other neurological condition were excluded. Inclusion and exclusion criteria were the same as in Dukic et al. [4].

### Clinical assessment and motor symptom scores

Motor impairment was graded by a lower motor neuron (LMN) score representing a sum of the Medical Research Council (MRC) muscle power scores in the following upper limb muscles: deltoid, triceps, biceps, wrist flexors and extensors, fingers flexors and extensors, first interosseous muscle, and abductor pollicis brevis; it ranged from 0 (severe LMN signs) to 90 (absent LMN signs) corresponding to 9 (muscles) × 2 (sides) × 5 (maximum MRC score) = 90. Clinical examination also included deep tendon reflexes of upper limbs and Hoffmann's Sign. The upper motor neuron (UMN) score was calculated by adding 1 point for each abnormal reflex observed ranging from 0 (absent UMN signs) to 8 (severe UMN signs) corresponding to (3 [brisk reflexes] + 1 [positive Hoffmann's Sign]) × 2 (sides) = 8.

Functional disability was assessed with the ALSFRS‐R. The fine motor function (FMF) subscore was calculated considering four items from the scale (handwriting, cutting food and handling utensils, dressing and hygiene, turning in bed and adjusting bed clothes). ALSFRS‐R progression rate was estimated as (48 − ALSFRS‐R score)/disease duration in months, and FMF progression rate was calculated as (16 − FMF subscore)/disease duration in months. All measures were evaluated on the day of EEG recording.

### EEG data acquisition and analysis

Six minutes of resting‐state eyes‐open EEG data were acquired using the Active Two system (BioSemi, Amsterdam, the Netherlands) at the Clinical Research Facility in St. James's Hospital, Dublin. Participants were seated in a chair and instructed to remain relaxed while they fixate their gaze at the letter X printed on a sheet of paper approximately 1 m away. Source localization was performed using the linearly constrained minimum‐variance beamformer to obtain time‐varying signals originating from the brain. An atlas‐based approach, based on the Automated anatomical labelling atlas, was used to estimate β‐band (14–30 Hz) spectral power in six regions: left/right precentral, postcentral, and paracentral areas. Spectral power was estimated using Fourier analysis, normalized using the total power of the filtered data (1–97 Hz), and averaged within the six sensorimotor regions. Further details are in Dukic et al [4].

### Statistical analysis

Differences in demographics between groups were calculated using the Mann–Whitney *U*‐test for age and Fisher exact test for sex distribution differences. The Shapiro–Wilk test was used to assess the normality of the EEG and clinical data distributions. As all variables had a normal distribution, the *t*‐test was used to assess the β‐band spectral power difference between the two groups and Pearson correlation was used to determine the relationship between β‐band spectral power and UMN, LMN, FMF subscore, and disease progression. The presented *p*‐values are after correction for multiple comparisons using false discovery rate (FDR; *q* = 0.05). Age did not demonstrate statistical significance as a covariate; thus, it was not included in the analysis.

## RESULTS

Eighteen people with ALS and 38 healthy controls were prospectively enrolled. Demographics and clinical characteristics are listed in Table 1.

**TABLE 1:** Demographics and clinical profiles.

Characteristic	ALS	Controls	*p*
*n*	18	38	
Age, years	65.75 [60.15–72.88]	66.00 [62.00–68.00]	0.60
Sex, male/female	12/6	22/16	0.57
Site of onset, upper spinal/lower spinal/bulbar/respiratory	3/11/3/1		
Disease duration, months	21.85 [18.6–27.8]		
ALSFRS‐R	36 [34–41]		
FMF subscore	12 [11–14]		
ALSFRS‐R progression rate, points/month	0.48 [0.26–0.7]		
FMF progression rate, points/month	0.2 [0.1–0.25]		
LMN score	78 [70–84]		
UMN score	2.5 [0–7]		
King stage, 1/2/3/4	4/6/3/5		

*Note*: Data are shown as median [interquartile range], except for *n*, sex, site of onset, and King stage, which are shown as counts. Disease duration is the time interval between the estimated symptom onset and the electroencephalographic recording. King staging assesses the disease burden in stages from 1 (single region involved) to 4 (ventilatory support and/or gastrostomy).

Abbreviations: ALS, amyotrophic lateral sclerosis; ALSFRS‐R, Amyotrophic Lateral Sclerosis Functional Rating Scale–Revised; FMF, fine motor function; LMN, lower motor neuron; UMN, upper motor neuron.

**FIGURE 1:**
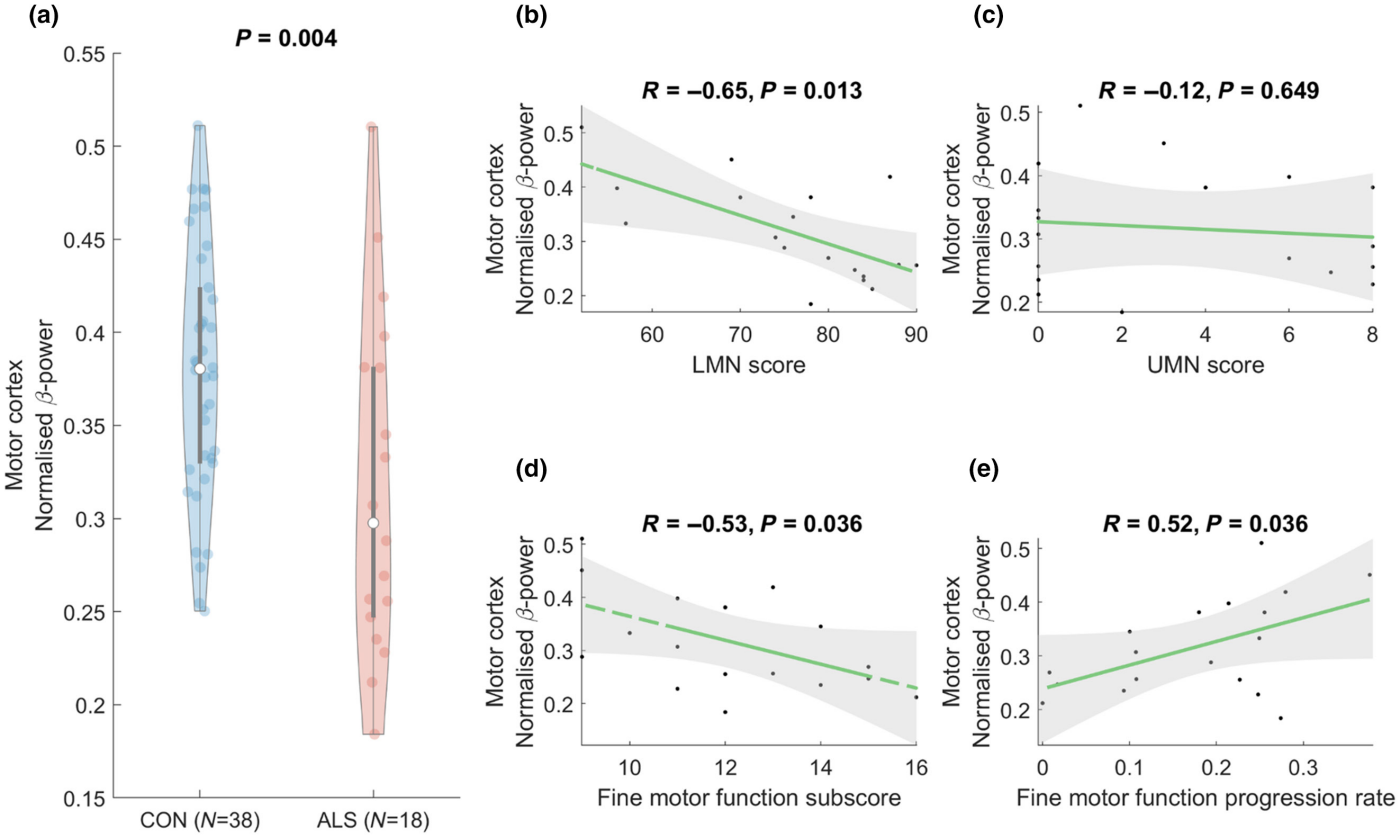
Normalized β‐band power in the sensorimotor network is decreased in amyotrophic lateral sclerosis (ALS) patients and shows correlation with the clinical measures of motor symptom severity (lower motor neuron [LMN] score and fine motor function subscore) and progression. The presented *p*‐values are after false discovery rate (*q* = 0.05) correction, and statistical significance is considered as *p* < 0.05. CON, control; UMN, upper motor neuron.

The β‐band power in the motor network was significantly lower in people with ALS compared to healthy participants (*p* = 0.004; Figure 1a). The β‐band power significantly correlated with motor impairment in people with ALS as measured by the LMN score (*R* = −0.65, *p* = 0.013; Figure 1b), where higher β‐power was associated with greater muscular weakness. Moreover, the β‐band power showed a significant correlation with the FMF subscore (*R* = −0.53, *p* = 0.036; Figure 1d) and the FMF progression rate (*R* = 0.52, *p* = 0.036; Figure 1e), where higher β‐power was associated with higher functional disability and faster disease progression. Although data might suggest that more severe UMN impairment was associated with lower β‐power, this correlation was not significant (Figure 1c).

## DISCUSSION

In this cross‐sectional study, we have shown that source‐localized β‐band power in the sensorimotor network reflects motor impairment and functional disability of people with ALS. These findings expand the previously described use of resting‐state EEG as a multidimensional tool for interrogating different networks and phenotyping people with ALS [4, 8].

Confirming the previous findings [4, 5], people with ALS had decreased source‐localized β‐band power in the sensorimotor network, supporting the hypothesis that β‐band oscillations are pathologically affected in ALS [10, 12]. Here, we have demonstrated a correlation between the β‐band power and disability in ALS. These findings suggest that β‐band power not only reflects cortical dysfunction but also the state of the entire motor system, encompassing UMNs, the spinal cord, LMNs, and peripheral nerves. This aligns with the notion that neural β‐band oscillations are influenced by corticospinal projections from the motor system to muscles, indicating the activity of both motor neurons and local interneurons [11].

At group level, people with ALS showed lower β‐band power compared to heathy controls, and we identified a trend towards a progressive increase with greater motor impairment in ALS. This result is in keeping with a recent study [9], which showed that the median β‐band power is higher in people with ALS compared to healthy controls, when participants with more severe clinical conditions and longer disease duration are considered. As reduction in β activity is associated with an activation of the motor cortex, our findings support the notion that cortical hyperexcitability is an early feature of ALS [13], which is progressively reduced over time [14], and could explain the increasing β‐band power in our results. Consistent with this, we have also shown that β‐band power correlates with ALS‐related progressive disability; patients with lower FMF subscore and higher rate of disease progression tended to have a higher β‐band power in the sensorimotor network. We did not observe a significant correlation with the UMN score, which could be attributed to the clinical assessment of UMN impairment being particularly challenging in patients with substantial LMN degeneration, thus warranting better markers of UMN signs [15].

This study is limited by the relatively small dataset. To achieve a more comprehensive understanding of the evolving patterns of cortical dysfunction in ALS, larger and longitudinal studies are essential, covering patients with a broad spectrum of disease durations including early stage. Additionally, we recognize that our analysis could have been strengthened with more extensive neurological examinations, encompassing both upper and lower limbs. Finally, it is important for future studies to establish links between cognitive/behavioural changes in ALS and EEG alterations.

In conclusion, our results show that β‐band power in the sensorimotor network has potential as an accessible and quantitative EEG‐based candidate biomarker of functional disability in ALS, which can be used along with other EEG‐based biomarkers of network dysfunction.

## FUNDING INFORMATION

Funding for this study was provided by the Health Research Board of Ireland (HRA‐POR‐2013‐246; MRCG‐2018‐02), Irish Motor Neurone Disease Research Foundation (IceBucket Award; MRCG‐2018‐02), Irish Research Council (Government of Ireland Postdoctoral Research Fellowship GOIPD/2015/213 to B.N. and Government of Ireland Postdoctoral Postgraduate Scholarship GOIPG/2017/1014 to R.M.), and Science Foundation Ireland (16/ERCD/3854). Data analytic aspects of the study were supported by the ALS Association (multiyear grant 20‐IIA‐546).

## CONFLICT OF INTEREST STATEMENT

The authors declare no conflicts of interest.

## Data Availability

The data that support the findings of this study are available on request from the corresponding author. The data are not publicly available due to privacy or ethical restrictions.
